# Metagenomics next-generation sequencing for the diagnosis of central nervous system infection: A systematic review and meta-analysis

**DOI:** 10.3389/fneur.2022.989280

**Published:** 2022-09-20

**Authors:** Chunrun Qu, Yu Chen, Yuzhen Ouyang, Weicheng Huang, Fangkun Liu, Luzhe Yan, Ruoyu Lu, Yu Zeng, Zhixiong Liu

**Affiliations:** ^1^Department of Neurosurgery, Xiangya Hospital, Central South University, Changsha, China; ^2^Xiangya School of Medicine, Central South University, Changsha, China; ^3^National Clinical Research Center for Geriatric Disorders, Xiangya Hospital, Central South University, Changsha, China

**Keywords:** metagenomic next-generation sequencing, central nervous system, infection, diagnosis, meta-analysis

## Abstract

**Objective:**

It is widely acknowledged that central nervous system (CNS) infection is a serious infectious disease accompanied by various complications. However, the accuracy of current detection methods is limited, leading to delayed diagnosis and treatment. In recent years, metagenomic next-generation sequencing (mNGS) has been increasingly adopted to improve the diagnostic yield. The present study sought to evaluate the value of mNGS in CNS infection diagnosis.

**Methods:**

Following the Preferred Reporting Items for Systematic reviews and Meta-Analyses (PRISMA) 2022 guidelines, we searched relevant articles published in seven databases, including PubMed, Web of Science, and Cochrane Library, published from January 2014 to January 2022. High-quality articles related to mNGS applications in the CNS infection diagnosis were included. The comparison between mNGS and the gold standard of CNS infection, such as culture, PCR or serology, and microscopy, was conducted to obtain true positive (TP), true negative (TN), false positive (FP), and false negative (FN) values, which were extracted for sensitivity and specificity calculation.

**Results:**

A total of 272 related studies were retrieved and strictly selected according to the inclusion and exclusion criteria. Finally, 12 studies were included for meta-analysis and the pooled sensitivity was 77% (95% CI: 70–82%, *I*^2^ = 39.69%) and specificity was 96% (95% CI: 93–98%, *I*^2^ = 72.07%). Although no significant heterogeneity in sensitivity was observed, a sub-group analysis was conducted based on the pathogen, region, age, and sample pretreatment method to ascertain potential confounders. The area under the curve (AUC) of the summary receiver operating characteristic curve (SROC) of mNGS for CNS infection was 0.91 (95% CI: 0.88–0.93). Besides, Deek's Funnel Plot Asymmetry Test indicated no publication bias in the included studies (**Figure 3**, *p* > 0.05).

**Conclusion:**

Overall, mNGS exhibits good sensitivity and specificity for diagnosing CNS infection and diagnostic performance during clinical application by assisting in identifying the pathogen. However, the efficacy remains inconsistent, warranting subsequent studies for further performance improvement during its clinical application.

**Study registration number:**

INPLASY202120002

## Introduction

Central nervous system (CNS) infection is caused by pathogens like bacteria, viruses, fungi, and parasites, including meningitis, encephalitis, and brain abscesses (1). It is a severe problem threatening human health, accounting for high mortality and morbidity worldwide. As a major global public health issue, up to 5 million cases are reported annually. One of the reasons for the high mortality is the difficult diagnosis of CNS infection and identification of the pathogens.

Most microbiology laboratories use traditional diagnostic techniques such as the isolation of microorganisms in culture, directed PCR, and serology. Nowadays, clinicians often rely on these traditional microbiologic methods, which are considered the gold standard for diagnosis but have significant limitations. Indeed, various pathogens that cause CNS infections are relatively rare, with few diagnostic assays available for them. Moreover, some traditional methods cannot distinguish between infectious and non-infectious inflammation, and the results of culture or PCR can be influenced by previous antibiotic exposure. Finally, in some cases, CNS infections may be caused by multiple infectious pathogens.

In recent years, a new method known as metagenomic next-generation sequencing (mNGS) has been increasingly used during clinical practice. Next-generation sequencing (NGS), also known as high-throughput sequencing, is characterized by high output and resolution, enabling sequence reads of large-scale DNA or RNA molecules in parallel in a single run, generating millions to billions of reads and providing a wealth of genetic information. An increasing body of evidence suggests that NGS has clinical significance for complex diseases, such as cancer. mNGS can not only comprehensively analyze the entire content of microbial and host genetic material (DNA or RNA) in patient samples but also greatly reduce the cost and time of sequencing (2). It also allows genomic characterization and identification of parasites, fungi, bacteria, and viruses, without prior knowledge of specific pathogens from clinical specimens (3). Albeit false-negative and false-positive results still exist, extensive pathogen detection and rapid diagnosis can be achieved using mNGS with high sensitivity and specificity (4). Furthermore, mNGS is indicated for identifying the etiology of unexplained or post-treatment infections (5–8). For a negative cerebrospinal fluid culture, mNGS can still be used to detect latent pathogens to assist in clinical diagnosis and prompt treatment (9). To summarize, mNGS is promising as an important tool for accurately diagnosing infectious diseases to achieve individualization of patient therapy and care (10, 11). However, there is a lack of studies with consistent results and high-quality evidence for mNGS application in CNS infection.

Hence, this meta-analysis was aimed at systematically analyzing and estimating the value of mNGS in diagnosing CNS infections. In addition, we discussed the advantages, limitations, and future development directions of clinical mNGS applications on CNS infectious diseases.

## Methods

### Design and registration

We conducted a systematic review and meta-analysis of the diagnostic accuracy of mNGS. The study protocol was registered on the International Platform of Registered Systematic Review and Meta-Analysis Protocols (INPLASY). The registration number is INPLASY202120002. This meta-analysis was conducted following the Preferred Reporting Items for Systematic Reviews and Meta-Analyses (PRISMA) 2022 guidelines (Supplementary Table 1)

### Search strategy

We comprehensively retrieved studies on mNGS and CNS infections published from January 2014 to January 2022 in MEDLINE, Web of Science, Cochrane Library, Embase, ClinicalTrials, Clinicalkey, and the Chinese Clinical Trial Registry. The search strategy was as follows: (metagenomic next-generation sequencing OR mNGS) AND (encephalitis OR meningitis OR cephalomeningitis OR Brain Abscess OR Toxoplasmosis, Cerebral OR Central Nervous System Bacterial Infections OR Lyme Neuroborreliosis OR Meningitis, Bacterial OR Neurosyphilis OR Tuberculosis, Central Nervous System OR Central Nervous System Fungal Infections OR Meningitis, Fungal OR Neuroaspergillosis OR Central Nervous System Parasitic Infections OR Central Nervous System Helminthiasis OR Central Nervous System Protozoal Infections OR Central Nervous System Viral Diseases OR Cerebral Ventriculitis OR Encephalitis, Viral OR Limbic Encephalitis OR Meningitis, Viral OR Meningoencephalitis OR Pseudorabies OR Encephalomyelitis OR Epidural Abscess OR Infectious Encephalitis OR Encephalitis, Viral OR Meningoencephalitis).

The specific search strategies carried out by two independent reviewers (QC and CY) for retrieval from the different databases are shown in Supplementary Table 2.

Studies were included based on the following criteria:

(1) Studies published from January 2014 to January 2022 (since the first publication reporting CNS infection diagnosis by mNGS was published in 2014).
(2) Experimental or observational studies.

Studies were excluded based on the following criteria:

(1) The abstract and full text are unavailable for quality evaluation.
(2) Studies not conducted on humans.
(3) Studies with an insufficient sample size for analysis (*n* > 20).
(4) Studies, whereby the samples analyzed, are not the clinical gold standard. Bacterial identification is not by culture; viral identification is not by PCR or serological test; fungi identification is not by culture or serological test; parasite identification is not by serological test or biopsy.
(5) The true positive (TP), false positive (FP), false negative (FN), and true negative (TN) values cannot be extracted from the study.
(6) The study sample only contains positive or negative cases with no details on specificity and sensitivity.
(7) Studies that only investigated the target pathogen mycobacterium tuberculosis.
(8) Studies where the tested sample is not blood or cerebrospinal fluid

### Quality evaluation

Review Manager 5.4 software was applied to conduct a literature quality evaluation for included studies based on the QUADAS-2 criterion (12). This evaluation tool involves four parts: (I) CNS infection patient selection; (II) conduction and interpretation of mNGS; (III) conduction and interpretation of reference standard; (IV) flow and timing. In every part, the answers to important questions can be “YES,” “NO,” and “Unclear.” Based on the answers to important questions, we evaluated “Risk of Bias” and “Applicability Concerns” with “Low,” “High,” and “Unclear” (Supplementary Table 3). After evaluation, we used STATA version 15.0 to generate Deek's funnel plot to evaluate whether publication bias existed in the included studies. Two investigators (QC and CY) completed the above work independently. Disagreements were resolved via a discussion with the third investigator (OY).

### Data selection and statistical analysis

We divided the final 12 studies into 25 sub-studies according to the type of pathogen being tested. From these 25 sub-studies, we extracted the name of the first author, the study's publication year, the sample source region, age, type of research, method of sample pretreatment, sequencing platform, sequencing depth, classification of the pathogen, the gold standard, and the result of the study (including the numbers of TP, FP, FN, and TN) (Supplementary Table 4). To ensure accuracy, two investigators (QC and CY) extracted information from the included articles independently. Discrepancies between the two investigators were resolved via a discussion with a third investigator (OY).

The sensitivity and specificity of mNGS detection were calculated with a confidence interval (CI) of 95%. *I*^2^ was used to evaluate the heterogeneity between studies and reference criteria. A bigger *I*^2^ value was associated with greater heterogeneity (13), and 50% was set as the threshold. In addition, we combined the positive likelihood ratio, negative likelihood ratio, and diagnostic odds ratio. Correspondingly, forest plots of all indicators were drawn. To further evaluate the efficacy of mNGS, the area under the curve (AUC) of summary receiver operating characteristic (SROC) was calculated. Deek's funnel plot was also generated to detect publication bias. Subgroup analysis was performed based on potential influencing factors. STATA version 15.0 and Modular Integrated Distributed Analysis System (MIDAS) modules were applied to analyze and conduct the meta-analysis. It has been established that MIDAS is a perfectly implemented command for the meta-analysis of diagnostic test accuracy (14).

## Results

### Characteristics of studies

**Figure 1:**
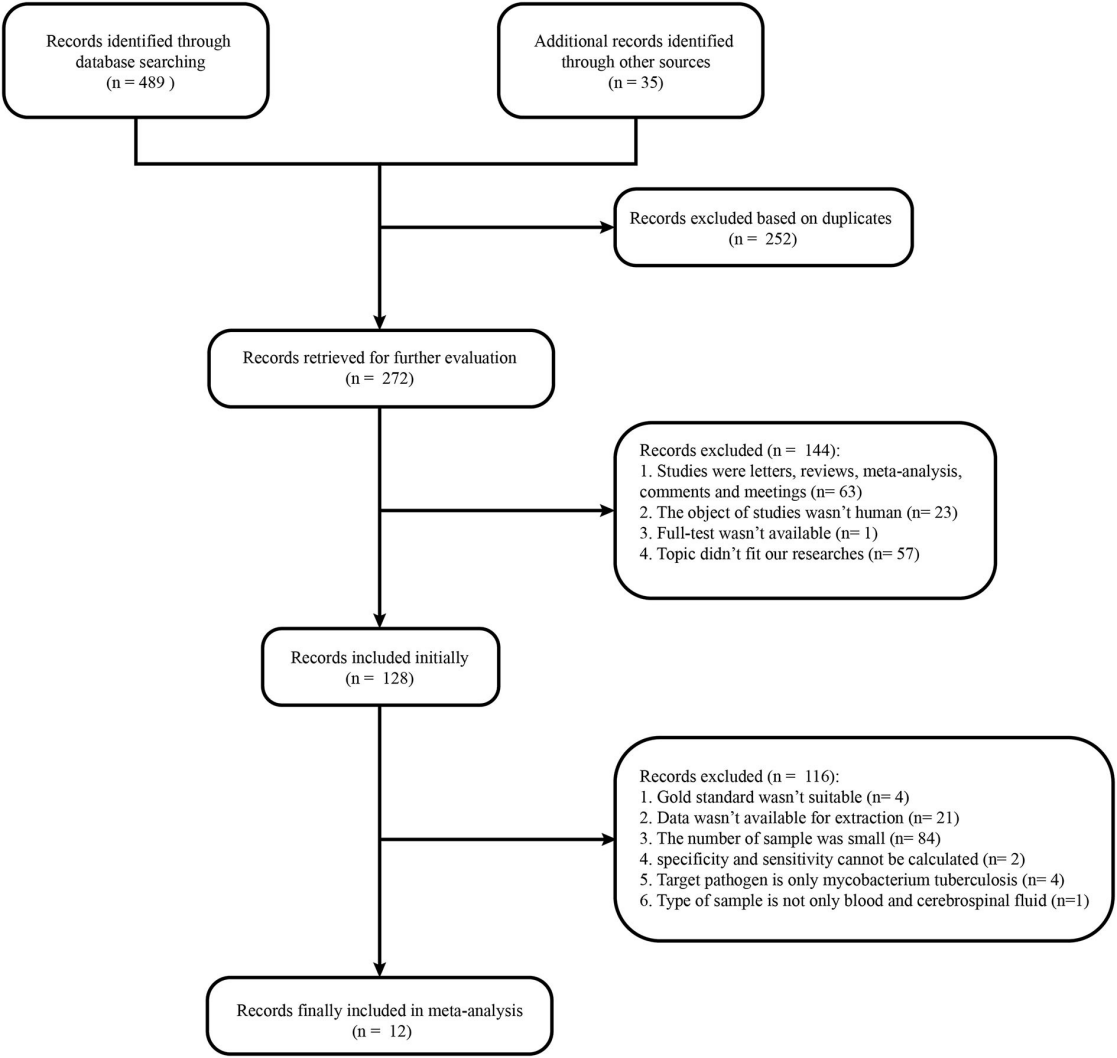
Flow chart of literature retrieval. In total, 165, 0, 147, 177, 18, 12, and 5 articles were found in PubMed, Cochrane Library, Embase, Web of Science, ClinicalKey, Chinese Clinical Trial Registry, and Clinical Trials, respectively.

The literature search yielded 272 studies from relevant databases, but only 12 were included in the final analysis (9, 15–25) (Figure 1). All included articles were in English. The 12 included studies were published between 2017 and 2021. Of which, four studies were retrospective (15, 16, 18, 20) and eight were prospective (9, 17, 19, 21–25). Studies were conducted in the following countries: China (9, 17, 22, 23), the United States (16, 18–21, 24), Canada (15), and the unknown (25). A total of 1,249 participants were analyzed in the 12 studies. The study with the smallest sample size had 37 participants (16), followed by 53 participants (22). Studies included participants in most age groups (child: <18 years old). The cerebrospinal fluid (CSF) was used for analysis in all included studies. Most studies were sequenced using Illumia (15–22, 24), but two studies were sequenced using the BGISEQ platform (9, 23), and one study could not identify the sequencing platform used (25). The pathogens examined included viruses (10/12), bacteria (12/12), fungi (2/12), and parasites (1/12). Among them, *Staphylococcus epidermidis, Streptococcus pneumoniae*, and *Klebsiella pneumoniae* were common bacteria in the mNGS diagnosis test, while herpes simplex virus and varicella zoster virus were common viruses. In terms of fungi and parasites, *Cryptococcus* and *Taenia Solium* were the only pathogens diagnosed in most cases. The 12 studies were initially divided into 25 sub-studies based on the detected pathogen. However, our meta-analysis was focused on 22 sub-studies because specificity and sensitivity from studies 16 and 18 could not be calculated and the sample size of study 15 did not satisfy our selection criteria.

### Study quality

**Figure 2:**
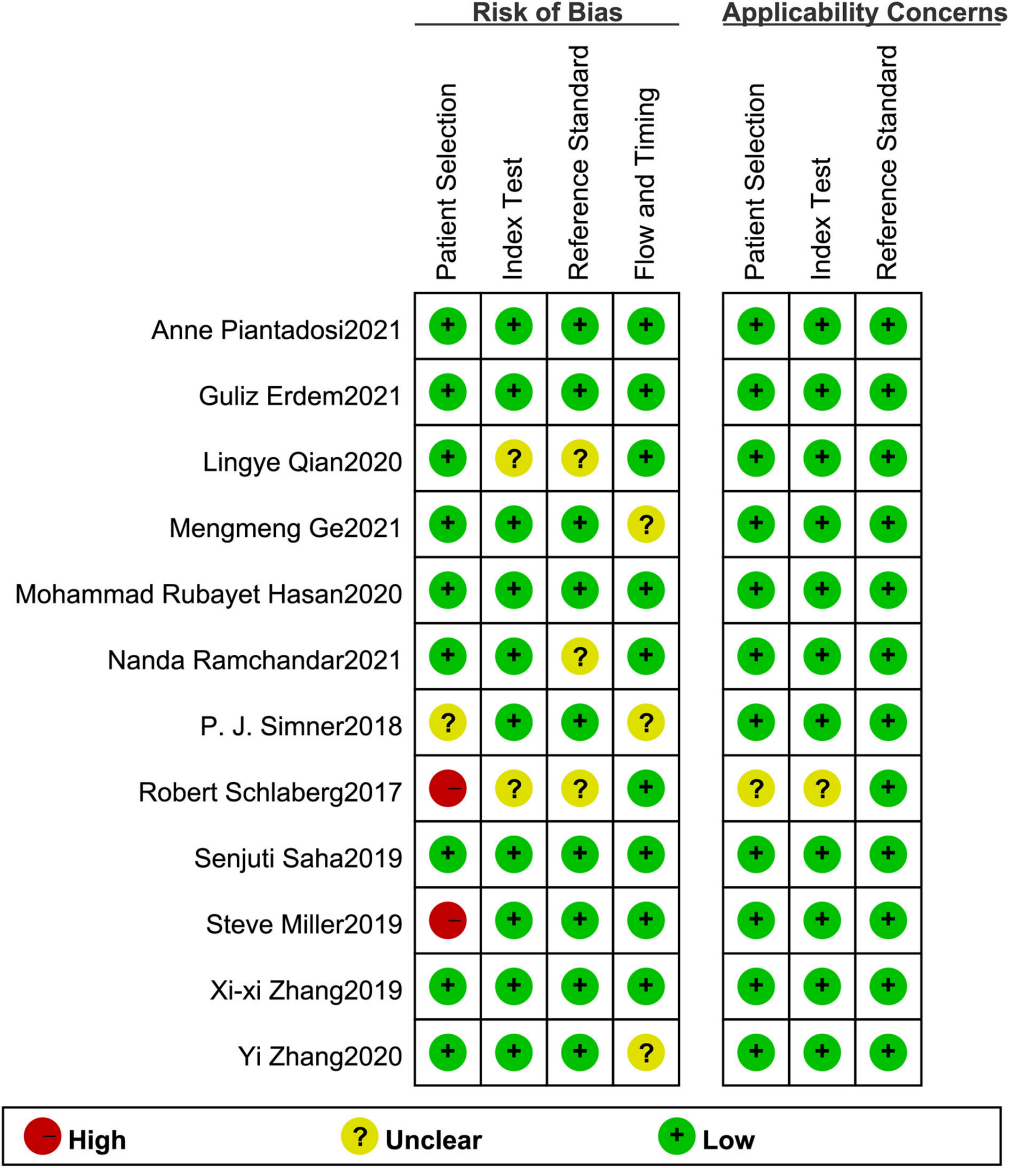
Quality assessment results of included studies based on QUADAS-2 tool criteria.

**Figure 3:**
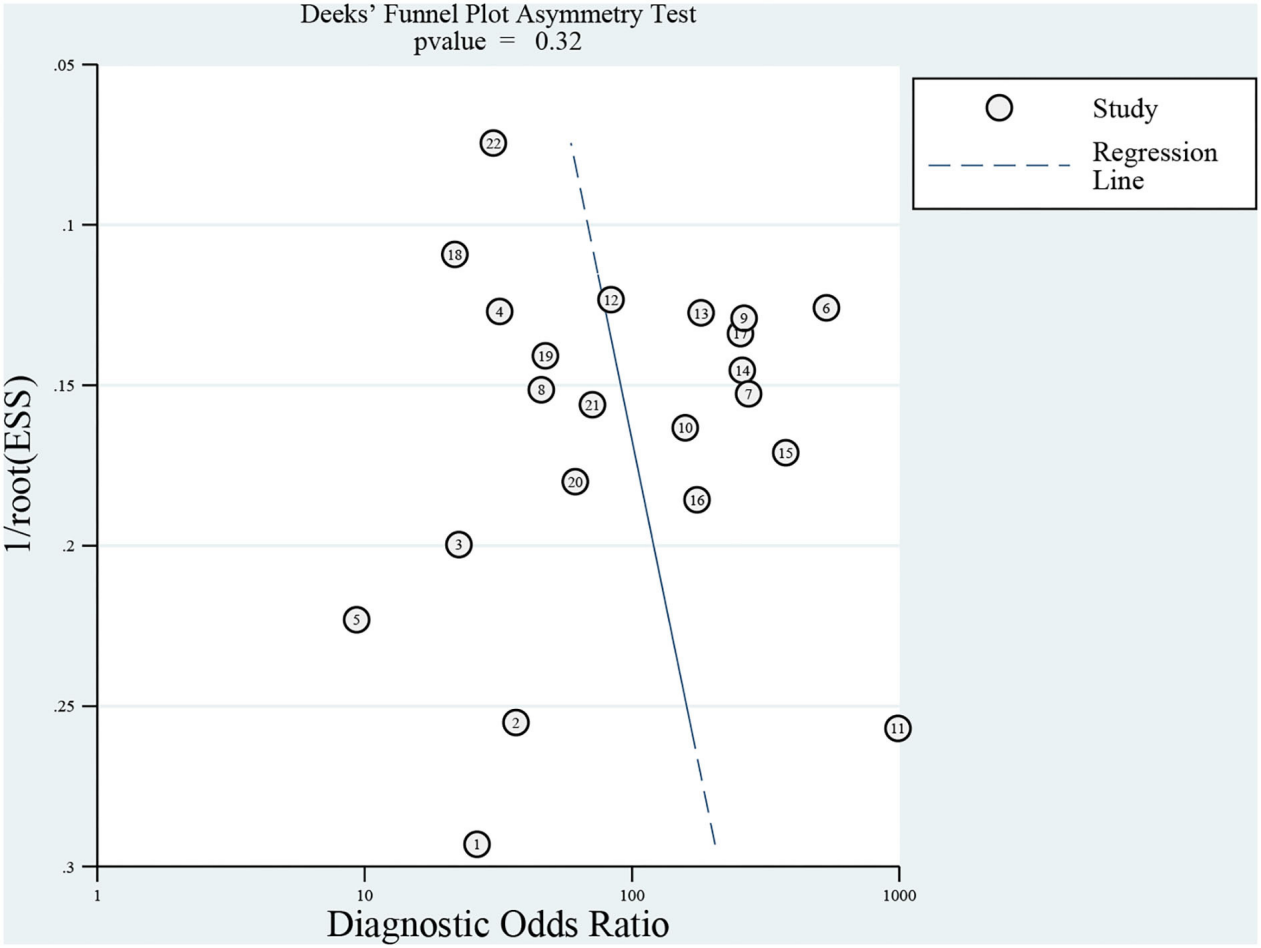
Funnel plot of publication bias.

The Review Manager 5.4 software was used to assess study quality. According to the result, most studies had good quality with low applicability concerns and a low risk of bias (Figure 2). Meanwhile, Deek's Funnel Plot Asymmetry Test indicated no publication bias in included studies (Figure 3; *p* > 0.05).

### Meta-analysis

**Figure 4:**
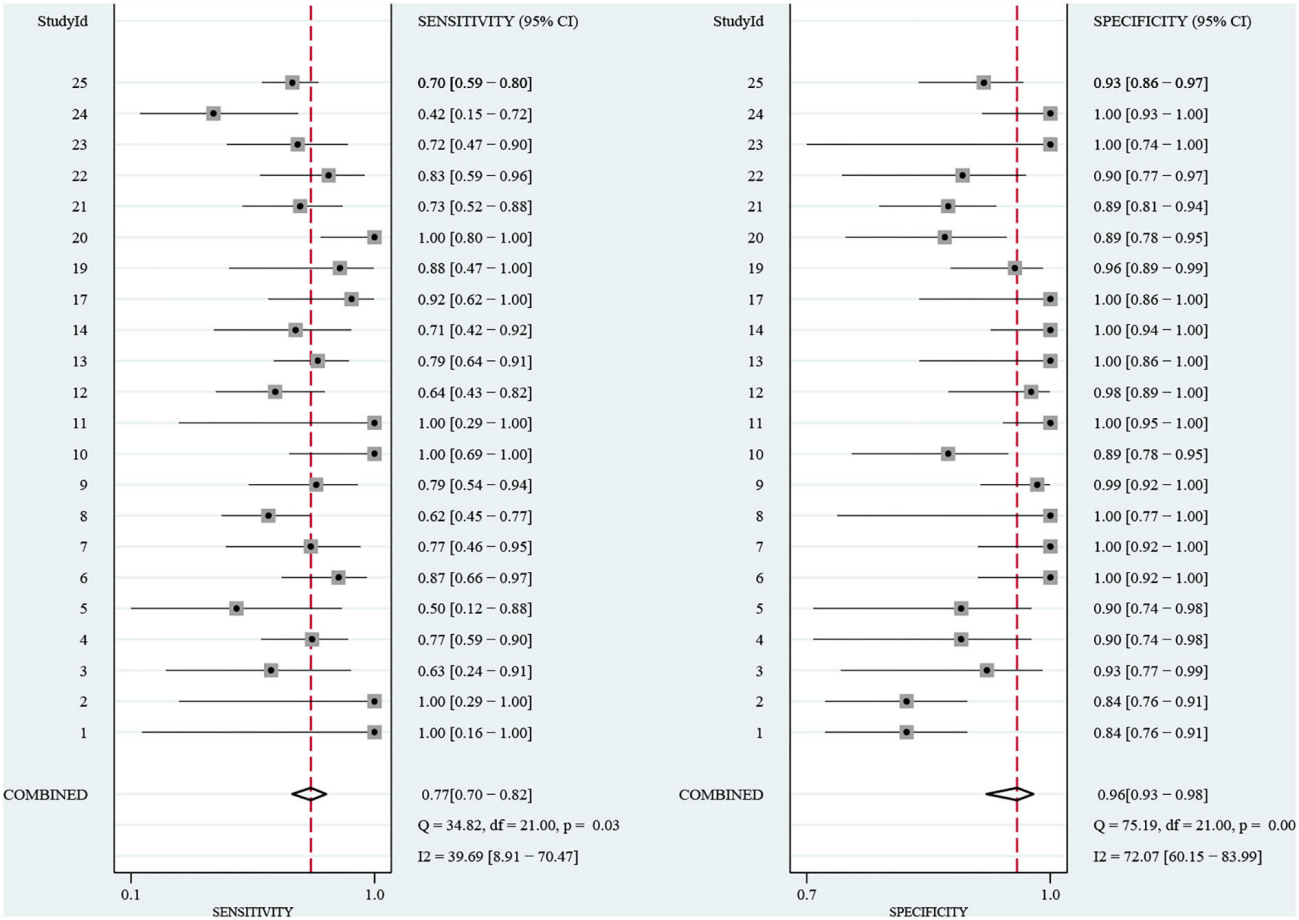
Forest plot for the sensitivity and specificity of mNGS for the diagnosis of CNS infection.

**Figure 5:**
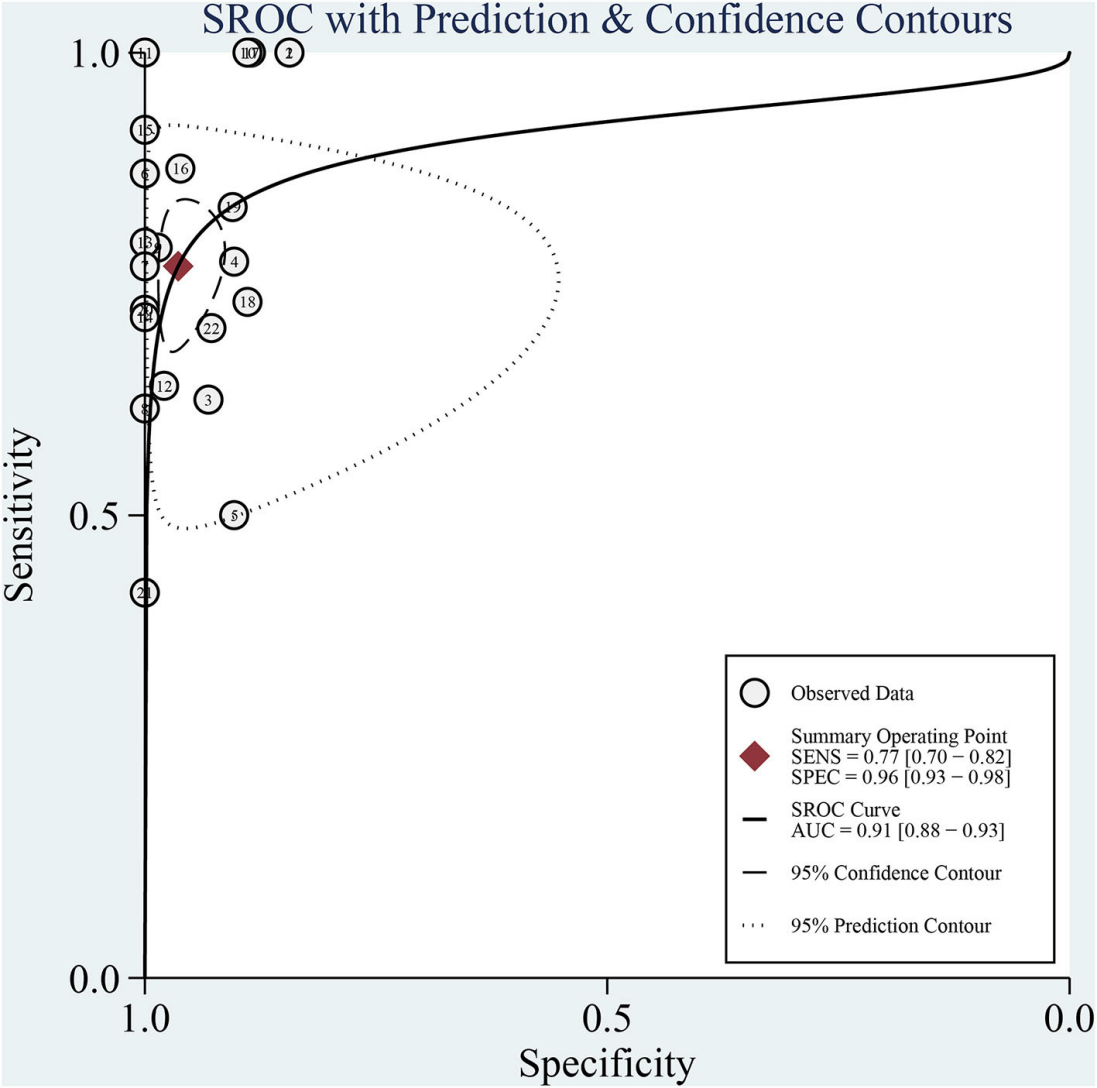
The SROC curve of mNGS diagnosis of CNS infection.

The sensitivity of mNGS for CNS infection ranged from 42 (95% CI: 15–72%) to 100% (95% CI: 80–100%). The combined sensitivity of mNGS for the diagnosis of CNS infection was 77% (95% CI: 70–82%), and the *I*^2^ value was 39.69% (95% CI: 8.91–70.47%). The specificity of mNGS ranged from 84 (95% CI: 76–91%) to 100% (95% CI: 93–100%) and the pooled specificity was 96% (95% CI: 93–98%), with an *I*^2^ value of 72.07% (95% CI: 60.15–83.99%) (Figure 4). Moreover, the pooled positive likelihood ratio, negative likelihood ratio, and diagnostic odds ratio were 21.32 (95% CI: 11.10–40.97, *I*^2^ = 35.99%), 0.24 (95% CI: 0.18–0.31, *I*^2^ = 37.36%), and 4.49 (95%CI: 3.79–5.19, *I*^2^ = 3.46%). More details of these indicators are displayed in Supplementary Figures 1, 2. Among the included studies, there is no significant heterogeneity in sensitivity, positive likelihood ratio, negative likelihood ratio, and diagnostic odds ratio. The SROC curve displayed the good performance of mNGS for diagnosing CNS infection. Figure 5 shows an AUC of 0.91 (95% CI: 0.88–0.93).

Due to the heterogeneity of our included articles in specificity based on the *I*^2^ value, subgroup analysis was conducted based on the pathogen, region, age, sample pretreatment method, and sequencing platform to explore potential confounding factors. Studies with accurate information on the parameter under investigation were included. Parameter levels for which the number of studies was insufficient for a statistically significant analysis were excluded from the analysis. Studies 5, 14, and 24 tested for pathogens that included microorganisms other than bacteria and viruses; however, the number of tests was insufficient to form separate groups during the subgroup analysis. Accordingly, these studies were not included in the subgroup analysis for pathogens. Moreover, studies 12, 13, 14, 22, 23, 24, and 25 did not specify the age composition of the participants and could not be included in the subgroup analysis for the age of the participants. Given that study 25 did not specify the areas in which the study was conducted, it was excluded from the subgroup analysis. Studies 1, 2, 3, 4, 5, 17, and 25 did not specify how the sample was pretreated and excluded from the subgroup analysis. Finally, 19, 15, 21, and 15 studies were included for subgroup analyses of pathogens, participants' age, region, and sample pretreatment methods, respectively. Illumina was used as the sequencing platform in most of the included studies, and there were very few studies using other sequencing platforms to analyze this factor. Subgroup analysis of the remaining factors indicated that the pooled effect of mNGS on bacterial identification was inferior to viral identification. Given the limited number of studies on fungi and parasites, no subgroup analysis was conducted. Meanwhile, we found that studies in non-Asian countries (North America) yielded better sensitivity and specificity than in Asian countries. In terms of age and sample pretreatment method, no significant difference existed in sensitivity and specificity, while mNGS on non-child populations yielded significantly better specificity than in child populations. More details are displayed in Table 1.

**Table 1:** Subgroup analysis results.

**Parameter**	**Category**	***N* studies**	**Sensitivity**	**p1**	**Specificity**	**p2**
Pathogen	Bacteria	10	0.77 [0.68–0.85]	0.00	0.95 [0.91–1.00]	0.03
	Virus	9	0.82 [0.75–0.90]		0.97 [0.93–1.00]	
	Child	7	0.86[0.76–0.96]	0.57	0.91 [0.86–0.97]	0.00
	Non-child	8	0.78 [0.68–0.89]		0.97 [0.95–1.00]	
Age	Asian country	5	0.75 [0.63–0.88]	0.03	0.93 [0.86–1.00]	0.00
	Non- Asian country (North America)	16	0.79 [0.72–0.85]		0.97 [0.95–0.99]	
Sample pre-treatment method	Fresh	6	0.84 [0.75–0.92]	0.41	0.97 [0.93–1.00]	0.56
	Frozen	9	0.73 [0.63–0.82]		0.99 [0.97–1.00]	

## Discussion

Central nervous system infection is a serious disease caused by various pathogens with serious complications, such as paralysis, coma, and even death. It is essential to adopt an appropriate diagnostic method to timely diagnose specific pathogens and improve patient survival. Over the past two decades, the diagnosis rates for CNS infection remained low despite the development of pathogen-specific testing, such as RT-PCR and antigen assays (26). However, the advent of sequencing technology mNGS provides a novel diagnostic approach for clinicians.

Herein, we collected data from prospective and retrospective studies. The pooled sensitivity and specificity of mNGS for the diagnosis of CNS infection were 77% (95% CI: 70–82%, *I*^2^ = 39.69%) and 96% (95% CI: 93–98%, *I*^2^ = 72.07%), respectively. The positive likelihood ratio, negative likelihood ratio, and diagnostic odds ratio were 21.32, 0.24, and 4.49, respectively, with an AUC of 0.91 (95% CI: 0.88–0.93), indicating an excellent diagnostic performance of mNGS for CNS infection. Due to the heterogeneity of our included articles in specificity based on the *I*^2^ value, sub-group analysis was conducted based on the detected pathogen, age, region, and sample pretreatment method. These four factors were identified as influencing factors of the specificity of mNGS diagnosis; the pathogen and region were also found as important factors influencing the sensitivity of mNGS results. The mNGS diagnostic performance of bacterial and viral infection was compared during the sub-group analysis of infectious pathogens. The diagnostic specificity and sensitivity of mNGS of viral infection were higher than for bacterial infection in the CNS. The relatively smaller genome of viruses compared to bacteria is probably an important factor in this phenomenon. During sub-group analysis based on age, mNGS exhibited higher specificity for adult CNS infection etiology identification than children. The relatively higher difficulty in obtaining samples from a child may hinder the better performance of mNGS clinical diagnosis. Interestingly, higher sensitivity and specificity were reported in North America than in Asia. The difference in mNGS technology availability, clinician proficiency, and patient race may influence the application of mNGS. Furthermore, the impact of sample pretreatment methods was assessed. Intriguingly, there was no significant difference in sensitivity and specificity between fresh and frozen samples, emphasizing that the diagnostic ability of mNGS is not limited by the storage method. Besides, sample type has been considered an important factor influencing the mNGS efficacy. However, CSF was analyzed in all included studies. Accordingly, we could not evaluate the difference among different sample categories.

By convention, all samples containing DNA or RNA to be tested are mixed and sequenced, then compared with the database. As promising high-throughput sequencing technologies, the main procedure of the mNGS technique constitutes multiple steps, including nucleic acid extraction, library preparation and sequencing, and bioinformatics analysis. With the advantages of hypothesis-free, culture-independent pathogen detection, a direct comparison between sequencing results and the pathogen genome database enables faster and more objective identification of infectious pathogens. Additionally, the impact of antibacterial drug use on the diagnostic yield of mNGS is less than in traditional culture (27). Of note, prior studies indicated that mNGS is beneficial for pathogen diagnosis during immunosuppressive host infection, and the positive rate of mNGS in viral and bacterial diagnosis is 3-fold higher than traditional methods (28). Besides, compared with bacterial culture, mNGS possesses the advantages of relatively short turnaround time, wide applicability, and high throughput capability. Importantly, mNGS is indicated for uncultivable bacteria, which often yield negative culture results. Chen et al. found that the detection rates of mNGS for bacterial and fungal infections were higher than the conventional culture method (95.0 vs. 60%) (29). Besides, the positive rate of the traditional culture method with cerebrospinal fluid is low, mostly <40% (9). Current evidence suggests that the sensitivity and specificity of culture are related to the concentration of bacteria, the use of antimicrobials, and the culture method (30). Interestingly, mNGS can also assist antibiotic use in appendicitis patients compared with bacterial culture (31). Compared with PCR, mNGS does not require pathogen identification and specific primer synthesis before the test, saving time for clinicians. In addition, parasites are commonly neglected pathogens of CNS infection. Microscopy, the current gold standard for parasite diagnosis, is often challenging and not suitable for rare parasite infection diagnoses, especially for young clinicians due to their lack of experience. For instance, Naegleria fowleri (*N. fowleri*) infection, which is rare in China, has been detected by mNGS in many cases (32–34). As for imaging examinations, such as CT or MRI, it is difficult to capture the imaging features of early inflammation and ascertain specific pathogens guiding drug use and therapy. To summarize, mNGS exhibits great potential for clinical application and possesses multiple advantages compared with traditional methods, highlighting that it has huge prospects for wide application in the diagnostic field (35).

The application of mNGS can be improved from the following aspects. First, the ability to interpret novel or rare mutations, detect structural gene variation, and copy number variation (CNV) is far from satisfactory (36). Although sequencing technology has been optimized in recent years, the lack of accessible resources such as databases for sequencing results interpretation still hinders wider clinical applications. It has been established that mNGS exhibits limited ability for structural rearrangement and CNV detection. However, numerous techniques and algorithms have been put forward, such as Sat-BSA (SVs associated with traits), CoverageMaster, and KNNCNV (K-Nearest Neighbor-based CNV detection) (37–39). Besides, the sensitivity and specificity of mNGS require further improvement, and combining mNGS with other assays is a promising strategy. For example, mNGS combined with conventional detection methods could increase the detection rate of *Mycobacterium tuberculosis* (40). The combination of mNGS results from multiple samples can be beneficial for clinical diagnosis in some cases. Although CSF is the optimal sample for mNGS detection, other sample types should be considered, such as blood and other body fluids (41). Repeated testing of samples is also recommended for better accuracy. In addition, the sequencing platform can influence mNGS results. Finally, given the relatively high cost of mNGS, the choice of mNGS for clinical infection diagnosis should also be researched from a health economics perspective. However, only a publication has hitherto compared the cost-effectiveness of mNGS and bacterial culture for periprosthetic joint infection (PJI) diagnosis. The cost-effectiveness analysis indicated that the mNGS should be considered when there is a high pretest probability of PJI compared with traditional bacterial culture (42). More cost-effectiveness or cost-benefit analyses are required to save costs and maximize benefits. Albeit these controversies, mNGS has been increasingly recommended for CNS infective pathogens identification by multiple expert consensuses in China (43, 44).

In our meta-analysis, different kinds of CNS infections, instead of a single disease, such as tubercular meningitis, were studied to comprehensively assess the diagnostic performance of mNGS. Besides, the sample size was sufficiently large to conduct further analyses, including sub-group analysis to exclude potential bias, heterogeneity, etc. Nonetheless, there were some limitations in the current study. First, we excluded studies with hard-to-extract data or studies where central nervous system infection diagnosis was not established. Hence, although biases may have affected the findings of this study to a certain extent, multiple analyses were conducted and substantiated the robustness of our results. Second, we excluded mycobacterium tuberculosis-related studies due to the poor efficacy of culture and PCR for diagnosing mycobacterium tuberculosis during clinical practice (45, 46). Therefore, such studies have no comparative value in our analysis. Meanwhile, the previous publication also excludes tuberculosis infection in their analysis (47). However, with the construction of reference standards in the future, mNGS on *Mycobacterium tuberculosis* diagnosis are worth exploring continuously.

## Conclusion

Overall, mNGS exhibits excellent sensitivity and specificity to establish the etiology of central nervous system infection, and the SROC curve indicated satisfactory diagnostic performance. The sub-group analysis showed that infectious pathogens, patient age, and region are potential influencing factors. Taken together, mNGS can be an important tool in diagnosing CNS infection and has great promise for clinical application. In addition, the most appropriate patient population should be identified and the entire process standardized from sample collection to data analysis to further improve its clinical efficacy.

## Data availability statement

The original contributions presented in the study are included in the article/supplementary material, further inquiries can be directed to the corresponding authors.

## Author contributions

CQ, YC, FL, ZL, and YZ: conception and design of the study. CQ, YC, YO, WH, FL, LY, and RL: preparation for the manuscript. CQ and YC: analysis and interpretation of data. All authors contributed to the article and approved the submitted version.

## Funding

The research was funded by the Natural Science Foundation of Hunan Province (2019JJ50961, 2022JJ70155), and the China Postdoctoral Science Foundation (2018M643006).

## Conflict of interest

The authors declare that the research was conducted in the absence of any commercial or financial relationships that could be construed as a potential conflict of interest.

## Publisher's note

All claims expressed in this article are solely those of the authors and do not necessarily represent those of their affiliated organizations, or those of the publisher, the editors and the reviewers. Any product that may be evaluated in this article, or claim that may be made by its manufacturer, is not guaranteed or endorsed by the publisher.

## Supplementary material

Supplementary Figure 1 Forest plot for positive likelihood ratio and negative likelihood ratio of mNGS for the diagnosis of CNS infection.

Supplementary Figure 2 Forest plot for diagnostic odds ratio of mNGS for the diagnosis of CNS infection.
