# Supplementary figures and images for: Metagenomics next-generation sequencing for the diagnosis of central nervous system infection: A systematic review and meta-analysis
Source: Front Neurol. 2022 Sep 20;13:989280. doi: 10.3389/fneur.2022.989280 (PMC9530978; doi:10.3389/fneur.2022.989280)

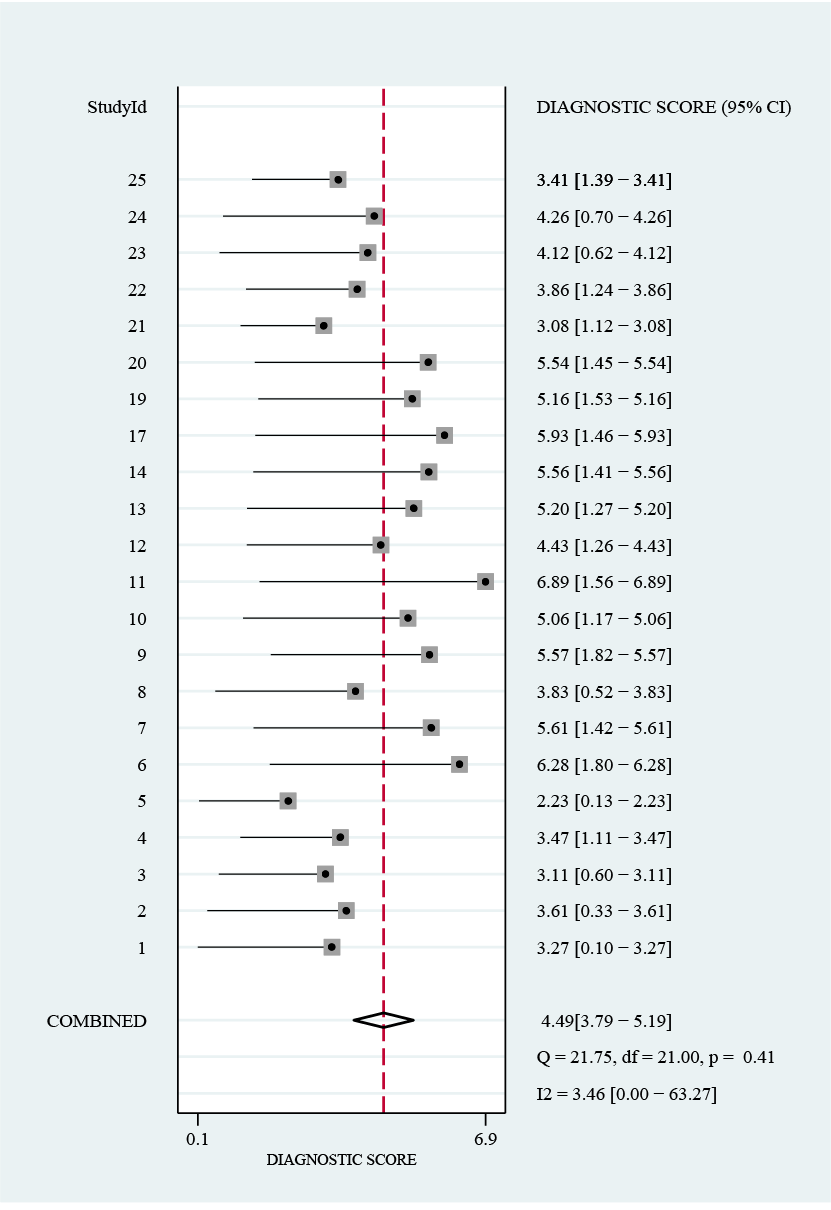

Supplement: Supplementary Figure 1 — Forest plot for positive likelihood ratio and negative likelihood ratio of mNGS for the diagnosis of CNS infection. [file Image_2.TIF]

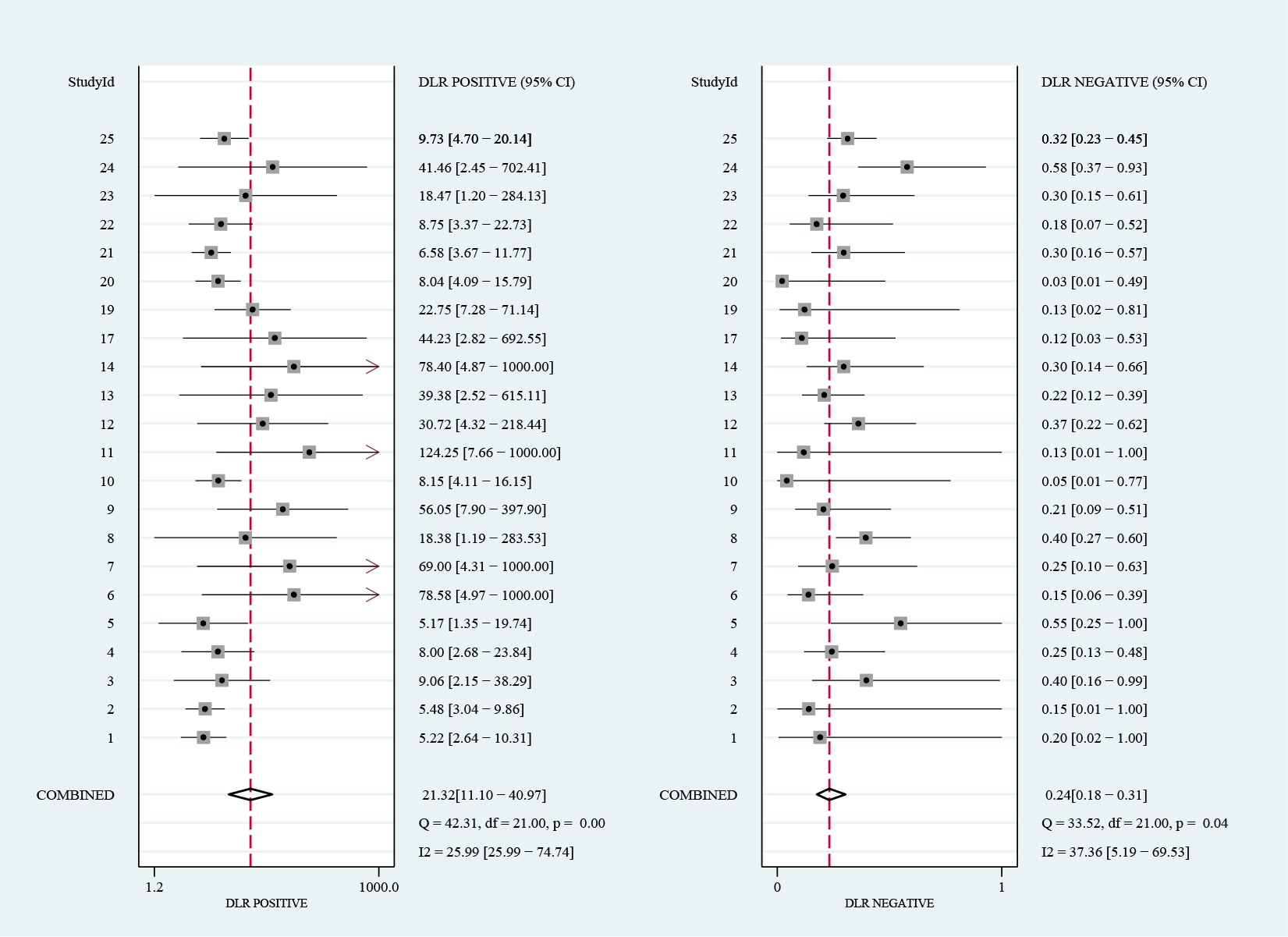

Supplement: Supplementary Figure 2 — Forest plot for diagnostic odds ratio of mNGS for the diagnosis of CNS infection. [file Image_1.TIF]
