# Supplementary material for: Metagenomics next-generation sequencing for the diagnosis of central nervous system infection: A systematic review and meta-analysis
Source: Front Neurol. 2022 Sep 20;13:989280. doi: 10.3389/fneur.2022.989280 (PMC9530978; doi:10.3389/fneur.2022.989280)
Supplement: Supplementary file 4 [file Table_2.DOCX]

**Supplementary table 2.** Search strategy

| Resource | Search terms | Number |
| --- | --- | --- |
| MEDLINE | (metagenomic next-generation sequencing OR "mNGS") AND ("encephalitis " OR "meningitis" OR "cephalomeningitis" OR "Brain Abscess" OR "Toxoplasmosis, Cerebral" OR "Central Nervous System Bacterial Infections" OR "Lyme Neuroborreliosis" OR "Meningitis, Bacterial" OR "Neurosyphilis" OR "Tuberculosis, Central Nervous System" OR "Central Nervous System Fungal Infections" OR "Meningitis, Fungal" OR "Neuroaspergillosis" OR "Central Nervous System Parasitic Infections" OR "Central Nervous System Helminthiasis " OR "Central Nervous System Protozoal Infections" OR "Central Nervous System Viral Diseases" OR "Cerebral Ventriculitis" OR "Encephalitis, Viral " OR "Limbic Encephalitis" OR "Meningitis, Viral " OR "Meningoencephalitis " OR "Pseudorabies" OR "Encephalomyelitis" OR "Epidural Abscess" OR "Infectious Encephalitis" OR "Encephalitis, Viral" OR "Meningoencephalitis" OR "Lupus Vasculitis, Central Nervous System" OR "Myelitis" OR "Perimeningeal Infections" OR "Epidural Abscess" OR "Subdural Effusion" OR "Anti-N-Methyl-D-Aspartate Receptor Encephalitis" OR "Encephalitis, Arbovirus" OR "Encephalitis, California" OR "Encephalitis, Japanese" OR "Encephalitis, St. Louis" OR "Encephalitis, Tick-Borne" OR "West Nile Fever" OR "Encephalitis, Herpes Simplex" OR "Encephalitis, Varicella Zoster" OR "Encephalomyelitis, Equine" OR "Encephalomyelitis, Eastern Equine" OR "Encephalomyelitis, Venezuelan Equine" OR "Encephalomyelitis, Western Equine" OR "Leukoencephalopathy, Progressive Multifocal" OR "Subacute Sclerosing Panencephalitis" OR "Arachnoiditis" OR "Meningitis, Aseptic" OR "Meningitis, Escherichia coli" OR "Meningitis, Haemophilus" OR "Meningitis, Listeria" OR "Meningitis, Meningococcal " OR "Meningitis, Pneumococcal" OR "Tuberculosis, Meningeal" OR "Meningitis, Cryptococcal" OR "Lymphocytic Choriomeningitis" OR "Waterhouse-Friderichsen Syndrome") | 165 |
| WEB OF SCIENCE | TS= (metagenomic next-generation sequencing OR "mNGS") AND TS= ("encephalitis " OR "meningitis" OR "cephalomeningitis" OR "Brain Abscess" OR "Toxoplasmosis, Cerebral" OR "Central Nervous System Bacterial Infections" OR "Lyme Neuroborreliosis" OR "Meningitis, Bacterial" OR "Neurosyphilis" OR "Tuberculosis, Central Nervous System" OR "Central Nervous System Fungal Infections" OR "Meningitis, Fungal" OR "Neuroaspergillosis" OR "Central Nervous System Parasitic Infections" OR "Central Nervous System Helminthiasis " OR "Central Nervous System Protozoal Infections" OR "Central Nervous System Viral Diseases" OR "Cerebral Ventriculitis" OR "Encephalitis, Viral " OR "Limbic Encephalitis" OR "Meningitis, Viral " OR "Meningoencephalitis " OR "Pseudorabies" OR "Encephalomyelitis" OR "Epidural Abscess" OR "Infectious Encephalitis" OR "Encephalitis, Viral" OR "Meningoencephalitis" OR "Lupus Vasculitis, Central Nervous System" OR "Myelitis" OR "Perimeningeal Infections" OR "Epidural Abscess" OR "Subdural Effusion" OR "Anti-N-Methyl-D-Aspartate Receptor Encephalitis" OR "Encephalitis, Arbovirus" OR "Encephalitis, California" OR "Encephalitis, Japanese" OR "Encephalitis, St. Louis" OR "Encephalitis, Tick-Borne" OR "West Nile Fever" OR "Encephalitis, Herpes Simplex" OR "Encephalitis, Varicella Zoster" OR "Encephalomyelitis, Equine" OR "Encephalomyelitis, Eastern Equine" OR "Encephalomyelitis, Venezuelan Equine" OR "Encephalomyelitis, Western Equine" OR "Leukoencephalopathy, Progressive Multifocal" OR "Subacute Sclerosing Panencephalitis" OR "Arachnoiditis" OR "Meningitis, Aseptic" OR "Meningitis, Escherichia coli" OR "Meningitis, Haemophilus" OR "Meningitis, Listeria" OR "Meningitis, Meningococcal " OR "Meningitis, Pneumococcal" OR "Tuberculosis, Meningeal" OR "Meningitis, Cryptococcal" OR "Lymphocytic Choriomeningitis" OR "Waterhouse-Friderichsen Syndrome") | 177 |
| COCHRANE LIBRARY | (metagenomic next-generation sequencing OR (mNGS)):ti,ab,kw AND ((encephalitis) OR (meningitis) OR (cephalomeningitis) OR (Brain Abscess) OR (Toxoplasmosis, Cerebral) OR (Central Nervous System Bacterial Infections) OR (Lyme Neuroborreliosis) OR (Meningitis, Bacterial) OR (Neurosyphilis) OR (Tuberculosis, Central Nervous System) OR (Central Nervous System Fungal Infections) OR (Meningitis, Fungal) OR (Neuroaspergillosis) OR (Central Nervous System Parasitic Infections) OR (Central Nervous System Helminthiasis) OR (Central Nervous System Protozoal Infections) OR (Central Nervous System Viral Diseases) OR (Cerebral Ventriculitis) OR (Encephalitis, Viral ) OR (Limbic Encephalitis) OR (Meningitis, Viral ) OR (Meningoencephalitis ) OR (Pseudorabies) OR (Encephalomyelitis) OR (Epidural Abscess) OR (Infectious Encephalitis) OR (Encephalitis, Viral) OR (Meningoencephalitis) OR (Lupus Vasculitis, Central Nervous System) OR (Myelitis) OR (Perimeningeal Infections) OR (Epidural Abscess) OR (Subdural Effusion) OR (Anti-N-Methyl-D-Aspartate Receptor Encephalitis) OR (Encephalitis, Arbovirus) OR (Encephalitis, California) OR (Encephalitis, Japanese) OR (Encephalitis, St. Louis) OR (Encephalitis, Tick-Borne) OR (West Nile Fever) OR (Encephalitis, Herpes Simplex) OR (Encephalitis, Varicella Zoster) OR (Encephalomyelitis, Equine) OR (Encephalomyelitis, Eastern Equine) OR (Encephalomyelitis, Venezuelan Equine) OR (Encephalomyelitis, Western Equine) OR (Leukoencephalopathy, Progressive Multifocal) OR (Subacute Sclerosing Panencephalitis) OR (Arachnoiditis) OR (Meningitis, Aseptic) OR (Meningitis, Escherichia coli) OR (Meningitis, Haemophilus) OR (Meningitis, Listeria) OR (Meningitis, Meningococcal) OR (Meningitis, Pneumococcal) OR (Tuberculosis, Meningeal) OR (Meningitis, Cryptococcal) OR (Lymphocytic Choriomeningitis) OR (Waterhouse-Friderichsen Syndrome)):ti,ab,kw | 0 |
| EMBASE | (metagenomic next-generation sequencing:ti,ab,kw OR 'mNGS':ti,ab,kw) AND ('encephalitis':ti,ab,kw OR 'meningitis':ti,ab,kw OR 'cephalomeningitis':ti,ab,kw OR 'brain abscess':ti,ab,kw OR 'toxoplasmosis, cerebral':ti,ab,kw OR 'central nervous system bacterial infections':ti,ab,kw OR 'lyme neuroborreliosis':ti,ab,kw OR 'meningitis, bacterial':ti,ab,kw OR 'neurosyphilis':ti,ab,kw OR 'tuberculosis, central nervous system':ti,ab,kw OR 'central nervous system fungal infections':ti,ab,kw OR 'meningitis, fungal':ti,ab,kw OR 'neuroaspergillosis':ti,ab,kw OR 'central nervous system parasitic infections':ti,ab,kw OR 'central nervous system helminthiasis':ti,ab,kw OR 'central nervous system protozoal infections':ti,ab,kw OR 'central nervous system viral diseases':ti,ab,kw OR 'cerebral ventriculitis':ti,ab,kw OR 'limbic encephalitis':ti,ab,kw OR 'meningitis, viral':ti,ab,kw OR 'pseudorabies':ti,ab,kw OR 'encephalomyelitis':ti,ab,kw OR 'infectious encephalitis':ti,ab,kw OR 'encephalitis, viral':ti,ab,kw OR 'meningoencephalitis':ti,ab,kw OR 'lupus vasculitis, central nervous system':ti,ab,kw OR 'myelitis':ti,ab,kw OR 'perimeningeal infections':ti,ab,kw OR 'epidural abscess':ti,ab,kw OR 'subdural effusion':ti,ab,kw OR 'anti-n-methyl-d-aspartate receptor encephalitis':ti,ab,kw OR 'encephalitis, arbovirus':ti,ab,kw OR 'encephalitis, california':ti,ab,kw OR 'encephalitis, japanese':ti,ab,kw OR 'encephalitis, st. louis':ti,ab,kw OR 'encephalitis, tick-borne':ti,ab,kw OR 'west nile fever':ti,ab,kw OR 'encephalitis, herpes simplex':ti,ab,kw OR 'encephalitis, varicella zoster':ti,ab,kw OR 'encephalomyelitis, equine':ti,ab,kw OR 'encephalomyelitis, eastern equine':ti,ab,kw OR 'encephalomyelitis, venezuelan equine':ti,ab,kw OR 'encephalomyelitis, western equine':ti,ab,kw OR 'leukoencephalopathy, progressive multifocal':ti,ab,kw OR 'subacute sclerosing panencephalitis':ti,ab,kw OR 'arachnoiditis':ti,ab,kw OR 'meningitis, aseptic':ti,ab,kw OR 'meningitis, escherichia coli':ti,ab,kw OR 'meningitis, haemophilus':ti,ab,kw OR 'meningitis, listeria':ti,ab,kw OR 'meningitis, meningococcal':ti,ab,kw OR 'meningitis, pneumococcal':ti,ab,kw OR 'tuberculosis, meningeal':ti,ab,kw OR 'meningitis, cryptococcal':ti,ab,kw OR 'lymphocytic choriomeningitis':ti,ab,kw OR 'waterhouse-friderichsen syndrome':ti,ab,kw) | 147 |
| CLINICAL TRIALS | metagenomic next generation sequencing OR "mNGS" \| Central Nervous System Infections | 5 |
| CLINICAL KEY | same as PubMed | 18 |
| CHINESE CLINICAL TRIAL REGISTRY | Public title is mNGS | 12 |
