# Supplementary material for: Metagenomics next-generation sequencing for the diagnosis of central nervous system infection: A systematic review and meta-analysis
Source: Front Neurol. 2022 Sep 20;13:989280. doi: 10.3389/fneur.2022.989280 (PMC9530978; doi:10.3389/fneur.2022.989280)
Supplement: Supplementary file 5 [file Table_3.DOCX]

**QUADAS-2 tools**

**Domain 1: Patient Selection**

*Risk of Bias: Could the Selection of Patients Have Introduced Bias?*

question 1: Was a consecutive or random sample of patients enrolled?

question 2: Was a case–control design avoided?

question 3: Did the study avoid inappropriate exclusions?

*Applicability: Are There Concerns That the Included Patients and Setting Do Not Match the Review Question?*

**Domain 2: Index Test**

*Risk of Bias: Could the Conduct or Interpretation of the Index Test Have Introduced Bias?*

question 1: Were the index test results interpreted without knowledge of the results of the reference standard?

question 2: If a threshold was used, was it prespecified?

*Applicability: Are There Concerns That the Index Test, Its Conduct, or Its Interpretation Differ From the Review Question?*

**Domain 3: Reference Standard**

*Risk of Bias: Could the Reference Standard, Its Conduct, or Its Interpretation Have Introduced Bias?*

question 1: Is the reference standard likely to correctly classify the target condition?

question 2: Were the reference standard results interpreted without knowledge of the results of the index test?

*Applicability: Are There Concerns That the Target Condition as Defined by the Reference Standard Does Not Match the Question?*

**Domain 4: Flow and Timing**

*Risk of Bias: Could the Patient Flow Have Introduced Bias?*

question 1: Was there an appropriate interval between the index test and reference standard?

question 2: Did all patients receive the same reference standard?

question 3: Were all patients included in the analysis?
