# Supplementary material for: Metagenomics next-generation sequencing for the diagnosis of central nervous system infection: A systematic review and meta-analysis
Source: Front Neurol. 2022 Sep 20;13:989280. doi: 10.3389/fneur.2022.989280 (PMC9530978; doi:10.3389/fneur.2022.989280)
Supplement: Supplementary file 6 [file Table_4.docx]

**Supplementary table 4.** Basic information of included literature

| **Study ID** | **Author** | **Year** | **Sample source region** | **Age** | **Type of research** | **Sample pre-treatment method** | **Sequencing platform** | **Sequencing depth** | **Gold standard** | **Classification** | **TP** | **FP** | **TN** | **FN** |
| --- | --- | --- | --- | --- | --- | --- | --- | --- | --- | --- | --- | --- | --- | --- |
| 1 | Mengmeng Ge | 2021 | Asian country (China) | child | prospective | unknown | Illumina | unknown | culture | Bacteria | 2 | 15 | 81 | 0 |
| 2 |  |  |  |  |  |  |  |  | PCR | virus | 3 | 15 | 81 | 0 |
| 3 | Guliz Erdem | 2021 | Non-Asian country (USA) | child | retrospective | unknown | unknown | unknown | PCR | virus | 5 | 2 | 27 | 3 |
| 4 | Anne Piantadosi | 2021 | Non-Asian country (USA) | non-child | prospective | unknown | Illumina | median depth of 6.19 × 10^6^ | PCR and serological test | virus | 24 | 3 | 28 | 7 |
| 5 |  |  |  |  |  |  |  |  | culture, PCR and serological test | bacteria; mycoplasma; spirochete | 3 | 3 | 28 | 3 |
| 6 | Nanda Ramchandar | 2021 | Non-Asian country (USA) | non-child | prospective | fresh | Illumina | median depth of 9.98 × 10^6^ | culture | bacteria | 20 | 0 | 45 | 3 |
| 7 |  |  |  |  |  |  |  |  | PCR and serological test | virus | 10 | 0 | 45 | 3 |
| 8 | Lingye Qian | 2020 | Asian country (China) | non-child | prospective | frozen | Illumina | unknown | culture | bacteria | 24 | 0 | 14 | 15 |
| 9 | Yi Zhang | 2020 | Asian country (China) | non-child | prospective | fresh | BGISEQ-100 | median depth of 1 | culture | bacteria | 15 | 1 | 70 | 4 |
| 10 | Mohammad Rubayet Hasan | 2020 | non-Asian country (Canada) | non-child | retrospective | frozen | Illumina | unknown | culture | bacteria | 10 | 7 | 56 | 0 |
| 11 |  |  |  |  |  |  |  |  | PCR | virus | 3 | 0 | 70 | 0 |
| 12 | Steve Miller* | 2019 | Non-Asian country (USA) | unknown | retrospective | frozen | Illumina | unknown | culture | bacteria | 16 | 1 | 47 | 9 |
| 13 |  |  |  |  |  |  |  |  | PCR and serological test | virus | 31 | 0 | 24 | 8 |
| 14 |  |  |  |  |  |  |  |  | culture and serological test | fungi | 10 | 0 | 55 | 4 |
| 15 | Steve Miller* | 2019 | Non-Asian country (USA) | child | prospective | unknown | Illumina | unknown | serological test | parasite | 1 | 0 | 4 | 0 |
| 16 |  |  |  |  |  |  |  |  | culture | bacteria | 0 | 2 | 18 | 0 |
| 17 |  |  |  |  |  |  |  |  | PCR and serological test | virus | 11 | 0 | 24 | 1 |
| 18 |  |  |  |  |  |  |  |  | culture and serological test | fungi | 0 | 0 | 4 | 0 |
| 19 | Senjuti Saha | 2019 | Non-Asian country (USA) | child | prospective | fresh | Illumina | average depth of 72×10^6^ reads/sample | culture | bacteria | 7 | 3 | 75 | 1 |
| 20 |  |  |  |  |  |  |  |  | PCR | virus | 17 | 7 | 54 | 0 |
| 21 | Xi-xi Zhang | 2019 | Asian country (China) | child | prospective | fresh | BGISEQ-500 | depth value of 1-36.57 | Culture | bacteria | 19 | 12 | 96 | 7 |
| 22 | Patricia J Simner | 2018 | Non-Asian country (USA) | unknown | retrospective | frozen | Illumina | 2.46-6.04 × 10^6^ | standard-of-care testing | bacteria | 15 | 4 | 38 | 3 |
| 23 |  |  |  |  |  |  |  |  | standard-of-care testing | virus | 13 | 0 | 12 | 5 |
| 24 |  |  |  |  |  |  |  |  | standard-of-care testing | fungi | 5 | 0 | 48 | 7 |
| 25 | Robert Schlaberg | 2017 | unknown | unknown | prospective | unknown | unknown | unknown | culture, PCR and serological test | virus, bacteria, fungi, and parasite | 59 | 7 | 90 | 25 |

* they are from the same study.
